# Confounding by Indication Probably Distorts the Relationship between Steroid Use and Cardiovascular Disease in Rheumatoid Arthritis: Results from a Prospective Cohort Study

**DOI:** 10.1371/journal.pone.0087965

**Published:** 2014-01-30

**Authors:** Alper M. van Sijl, Maarten Boers, Alexandre E. Voskuyl, Michael T. Nurmohamed

**Affiliations:** Department of Rheumatology, VU University Medical Center, Amsterdam, the Netherlands; Keio University School of Medicine, Japan

## Abstract

**Objective:**

To evaluate the risk of cardiovascular disease in patients with rheumatoid arthritis exposed to glucocorticoids.

**Methods:**

Retrospective analysis of exposure to glucocorticoids in a prospective cohort of 353 patients with rheumatoid arthritis followed from June 2001 up to November 2011 for incident cardiovascular disease in a hospital-based outpatient cohort in the Netherlands. Hazard ratios with 95%-confidence intervals were calculated for the association between different types of exposure to glucocorticoids and incident cardiovascular disease. Associations were adjusted for demographics, cardiovascular risk factors and disease related parameters.

**Results:**

Recent and current exposure to glucocorticoids were associated with incident cardiovascular disease, as was a longer duration of exposure and cumulative exposure to glucocorticoids. Adjustment for disease activity and severity negated the association.

**Conclusion:**

In observational studies the finding of incident cardiovascular disease in patients with rheumatoid arthritis exposed to glucocorticoids is strongly confounded by indication due to high disease activity. The adverse cardiovascular effects of glucocorticoids might be balanced by positive effects working through inflammation control.

## Introduction

Glucocorticoids (GC) are one of the most effective anti-rheumatic drugs, but concerns over long-term side effects have not been resolved. [1] In rheumatoid arthritis (RA), GC are disease-modifying agents as they rapidly provide symptomatic relief, suppress disease activity and, slow progression of radiological damage. [2], [3] However, recent studies suggest that cumulative exposure to GCs is associated with an increased risk of cardiovascular (CV) disease, perhaps in part through worsening of the CV risk profile. [4], [5].

Considering the systemic inflammatory process in RA, also responsible for the increased CV risk in this group, GC use might enhance or mitigate the pre-existing CV risk. The net effect of GC use on the risk of developing CV disease therefore remains elusive.

## Materials and Methods

A total of 353 individuals were followed in the prospective hospital-based outpatient cohort of the CArdiovascular Risk in patients with RhEumatoid arthritis (CARRÉ) study. The purpose of the CARRÉ study is to investigate CV disease and its risk factors in RA patients. [6] In 2000, a random sample of RA patients registered at the Jan van Breemen Research Institute - Reade in Amsterdam, the Netherlands, was drawn. Patients fulfilled the 1987 American College of Rheumatology classification criteria for RA, and were aged between 50 and 75 years. The local ethics committees and institutional review board of the VU University Medical Center in Amsterdam, the Netherlands, approved the study protocol and all participants gave their written informed consent for the study in accordance to the declaration of Helsinki.

At inclusion, prior medication use, baseline disease activity and CV risk factors were assessed. From clinical notes we collected data on GC exposure at baseline by ascertaining the duration of exposure, last exposure by date, mean dose of GC and cumulative exposure in grams. GC exposure was calculated as prednisone equivalents. Individuals, aged 50–75 years with a diagnosis of RA, were followed for approximately 10 years for the development of CV disease. Follow-up duration was calculated as time from enrolment in the study until follow-up measurement or occurrence of a (non-) fatal CV event. During the follow-up period, all CV events were recorded and adjudication of CV events was performed on the basis of standardized criteria by an independent trained person according to the ICD-9 codes for myocardial infarction (MI) (410.0–9), stroke (436) or transient ischemic attack (TIA) (435.9), a history of peripheral arterial reconstruction, carotid endarterectomy, percutaneous coronary intervention (PCI) (8036), coronary artery by-pass surgery (CABG) (8038) and sudden death, cause unknown (798). Data on the participants’ vital status on December 1, 2011 were collected from the local municipalities and general practitioners. For each subject, we determined whether or not an incident CV disease had occurred. If a patient had moved out of town, information on vital status was obtained from the new local municipalities. Information on health status could not be obtained for 31 (8.8%) participants.

The association between GC exposure at baseline and incident CV disease was analyzed using Cox proportional hazard analyses. Multivariate Cox proportional hazard analyses assessed whether this association was independent of: 1. age, gender, and 10-year estimated CV risk according to SCORE, and 2. age, gender, SCORE, Disease activity score in 28 joints (DAS-28), and the Health Assessment Questionnaire – Disability Index (HAQ-DI). Possible confounders were considered according to available literature and by selecting variables on the basis of the effect on the original estimate of the exposure effect. Results are described as hazard ratios (HR) with 95%-confidence intervals (95%-CI) expressing the risk of a CV event. Analyses were performed again with steroid exposure during follow-up period as independent variables, adjusting for demographic factors, CV risk, cumulative DAS28, cumulative HAQ and steroid use at baseline. Two sided p-values less than 0.05 were considered statistically significant. All analyses were performed by SPSS 17.0 (Chicago, IL, USA).

## Results

The study comprised of 353 individuals of whom 59 (17%) were on GC treatment at the time of inclusion. During the study period 95 (27%) individuals used GC at any given moment during follow-up. The median (interquartile range) duration of exposure of patients on GC before inclusion was 2.2 (0.6–4.8) years and the cumulative exposure before inclusion was 8.7 grams (2.5–25.4). After a total follow-up experience of 2.361 years, 58 (16.5%) participants developed an incident CV disease, yielding an incidence rate of 24.6/1.000 patient years.

**Figure 1:**
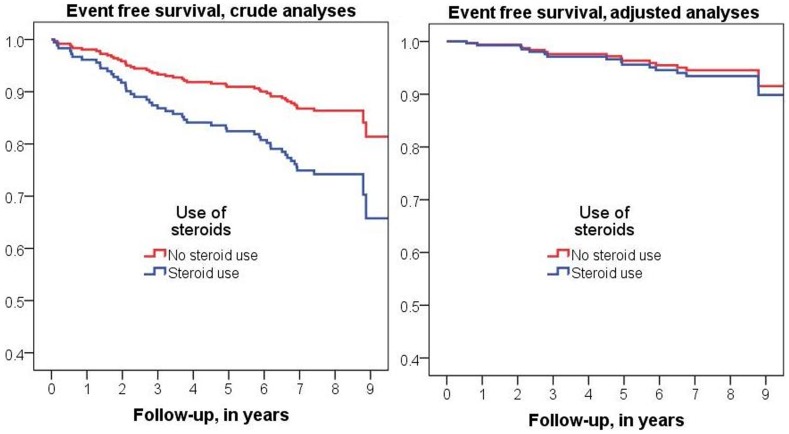
Kaplan-Meier survival curve of individuals who were using steroids at baseline vs. individuals who were not using steroids at baseline, both crude as well as fully adjusted for disease activity−/severity.

Compared to patients without, cases with incident CV disease included significantly more GC users at baseline, were treated with GC for a longer period and at higher cumulative doses. The associations remained similar after adjustment for age, gender and SCORE-estimated CV risk at baseline (Table 1: crude estimate and model 1, respectively). However, additional adjustment for disease activity (28-joint disease activity score, DAS28) and disability (Health Assessment Questionnaire – Disability Index (HAQ-DI), which is a measure that reflects both current inflammatory activity and the effects of cumulative joint damage) completely erased the associations of GC with incident CV disease (Figure 1). The standard error estimates of the regression coefficients did not increase substantially after correction for covariates, suggesting robustness of our observations. We can therefore assume the effect of GC remained present in most individuals at risk during follow-up. In addition, statistical analyses performed with prospective data on steroid use during follow-up period, with adjustment for cumulative DAS28 and HAQ, yielded essentially similar results, albeit in this analysis the results were not statistically significant.(data not shown) Furthermore, subgroup-analysis for patients who were not on concomittant NSAIDs did not reveal essentially different results.(data not shown).

**Table 1:** Cox-proportional hazard analyses of exposure to glucocorticoids at baseline and incident CV disease in patients with rheumatoid arthritis.

	Crude	Adjusted model 1¶	Adjusted model 2§
Steroid use			
- Ever vs. never	1.68 (0.92–3.07)	1.77 (0.68–4.63)	0.89 (0.26–3.09)
- Recent (<1 year) vs. never	1.93 (1.00–3.72)*	2.03 (0.72–5.74)	1.11 (0.27–4.53)
- Current vs. never	2.18 (1.12–4.27)*	2.91 (1.06–8.00)*	1.34 (0.31–5.88)
Duration of steroids, years	1.15 (1.01–1.31)*	1.18 (0.90–1.41)	1.14 (0.83–1.58)
- No steroids	1.00 (ref)	1.00 (ref)	1.00 (ref)
- = <5 years	1.00 (0.46–2.19)	1.02 (0.28–3.67)	0.71 (0.15–3.27)
- >5 years	4.64 (2.12–10.14)*	4.33 (1.31–14.29)*	1.48 (0.21–10.45)
Cumulative steroid use, grams	1.02 (1.01–1.04)*	1.04 (1.01–1.07)*	1.05 (0.99–1.11)
- No steroids	1.00 (ref)	1.00 (ref)	1.00 (ref)
- = <10 gram	0.92 (0.36–2.37)	0.43 (0.06–3.33)	0.42 (0.05–3.30)
- >10 gram	2.34 (1.15–4.77)*	3.88 (1.39–3.33)*	1.80 (0.37–8.74)
- No steroids	1.00 (ref)	1.00 (ref)	1.00 (ref)
- 1^st^ tertile	1.53 (0.60–3.92)	0.59 (0.08–4.67)	0.54 (0.07–4.36)
- 3^rd^ tertile	3.52 (1.72–7.18)*	5.21 (1.79–15.17)*	2.420.45–13.00)

* p<0.05. Results presented as hazard ratios (95%-confidence interval).

¶ Correction for age, gender, and 10-year estimated CV risk according to SCORE.

§ Correction for age, gender, SCORE, Disease activity score in 28 joints (DAS-28), and the Health Assessment Questionnaire – Disability Index (HAQ-DI).

## Discussion

We hypothesize that GC use in uncontrolled populations represents a marker of high RA disease activity; high disease activity itself represents an increased risk of CV disease. Thus the observed relationship of GC with CV disease in RA patients is strongly confounded by indication. Intensive treatment of systemic inflammation and disease activity remains an essential part of treatment of RA. In this setting, it is possible that the adverse CV effects of GC are balanced by positive effects working through inflammation control.

Prior studies found an increased risk of CV disease in patients who used steroids. A nested-case control study from a general practice database showed that individuals who currently use steroids were at increased risk of developing cardiovascular disease, cerebrovascular disease or heart failure. These results remained similar for patients with a diagnosis of RA and chronic obstructive pulmonary disease (COPD). A dose-response association with higher cumulative doses was not found. [7] In addition, two retrospective population cohort studies investigated the incidence of CV disease in patients with RA who used steroids. [4], [5] Both studies found an up to threefold increased risk of CV events in patients with RA who used steroids, especially in patients with rheumatoid factor positivity, higher cumulative exposure to steroids and exposure to steroids for more than 5 years. One of these studies even found that the use of low-dose glucocorticoids was associated with the development of a CV event. [5].

The present study confirms these findings, as we also found an increased risk of CV events in RA patients treated currently with steroids, patients treated for more than 5 years and patients treated with high cumulative doses. With additional adjustment for demographic parameters and cardiovascular risk, the association remained similar. However, when adjusted for disease activity and disability, the association is negated, thus reflecting an effect confounded by indication.

Previously, the relationship between steroid use and CV disease was always thought to be an unfavourable one. This study shows that this association is strongly mitigated by the disease activity and disability of the disease itself. The current guidelines on CV risk in rheumatoid arthritis state that “an adequate disease activity control is necessary to lower the CV risk”, while “glucocorticoids should be used in the lowest dose possible”. [8] If we extrapolate the findings from this study to the clinical situation that physicians and patients face on a daily basis, we would recommend that glucocorticoid use should be limited to small dosages and short periods of exposure. However, in patients with active RA, our data support the use of glucocorticoids to gain rapid disease control.

Strengths and weaknesses of this study merit careful consideration. Strengths of the present study include the recording of CV- and RA-related factors as well as medication use, which were all performed by three physicians using the same protocol. In addition, this study provides a well-defined study population with a long-term follow-up period that can investigate the effects of medication use on incident CV disease. However, our study has several limitations that should be acknowledged. First, we did not use a control population of healthy subjects and therefore were not able to compare the present findings in RA-patients with matched non-RA patients. Second, small number of incident cases and soft events (elective PCI or CABG) might have caused a relatively low power. Therefore, we restricted confounding analyses to a minimum of variables. Third, relatively diminished use of steroids in the follow-up period limited our power for analyses of prospective steroid use and the risk of incident CV disease.

In conclusion, this study suggests that glucocortioid use is a risk factor for CV events in RA, but that this association is strongly confounded by disease activity and disability.
